# Dietary anthocyanin intake and age-related decline in lung function: longitudinal findings from the VA Normative Aging Study^1^,^2^,^3^

**DOI:** 10.3945/ajcn.115.121467

**Published:** 2016-01-20

**Authors:** Amar J Mehta, Aedín Cassidy, Augusto A Litonjua, David Sparrow, Pantel Vokonas, Joel Schwartz

**Affiliations:** 4 Department of Environmental Health, Harvard School of Public Health, Boston, MA;; 5 Department of Nutrition, Norwich Medical School, University of East Anglia, Norwich, United Kingdom;; 6 Channing Division of Network Medicine and; 7 Division of Pulmonary and Critical Care Medicine, Brigham and Women's Hospital, Harvard Medical School, Boston, MA;; 8 The VA Normative Aging Study, VA Boston Healthcare System, Boston, MA; and; 9 Department of Medicine, Boston University School of Medicine, Boston, MA

**Keywords:** anthocyanins, clinical epidemiology, diet, flavonoids, lung function tests

## Abstract

**Background:** It is unknown whether habitual intake of dietary flavonoids, known for their antioxidative and anti-inflammatory properties, affects longitudinal change in lung function.

**Objective:** We investigated whether different flavonoid subclasses present in the habitual diet were associated with beneficial changes in lung function over time in the elderly.

**Design:** This longitudinal analysis included 839 participants from the VA (Veterans Affairs) Normative Aging Study whose lung function [forced expiratory volume in 1 s (FEV_1_) and forced vital capacity (FVC)] was measured at 2 and up to 5 visits between 1992 and 2008 (*n* = 2623 measurements). Yearly average intake of major flavonoid subclasses (anthocyanins, flavanones, flavan-3-ols, flavonols, flavones, and polymers) was calculated from food-frequency questionnaires at each visit. We estimated adjusted differences in annual change in lung function associated with each flavonoid subclass, categorized into quartiles, in linear mixed-effects regression models after adjustment for lifestyle and dietary confounders.

**Results:** Strong inverse associations were found between anthocyanin intake and age-related decline in lung function. Independent of dietary and nondietary risk factors, slower rates of FEV_1_ and FVC decline by 23.6 (95% CI: 16.6, 30.7) and 37.3 (95% CI: 27.8, 46.8) mL/y, respectively, were observed in participants in the fourth quartile of intake compared with participants in the first quartile (*P*-trend < 0.0001). The protective associations observed for anthocyanin intake were present in both current/former and never smokers. Compared with no or very low intakes, an intake of ≥2 servings of anthocyanin-rich blueberries/wk was associated with slower decline in FEV_1_ and FVC by 22.5 (95% CI: 10.8, 34.2) and 37.9 (95% CI: 22.1, 53.7) mL/y, respectively. To a lesser extent, higher flavan-3-ol intake was also associated with slower lung function decline.

**Conclusions:** An attenuation of age-related lung function decline was associated with higher dietary anthocyanin intake in this longitudinal sample of predominantly elderly men. Further prospective studies are needed to confirm these novel associations.

## INTRODUCTION

Lung function starts to decline in the third decade of life (1), but with different rates of decline across individuals (2). Lower lung function is a strong predictor of mortality (3, 4), and faster lung function decline has been associated with increased risks of hospitalizations related to chronic obstructive pulmonary disease (5), a leading cause of mortality in all countries (6). There are multiple sources of the heterogeneity in lung function decline in adults, and the available evidence from population-based studies suggests that a diet rich in antioxidants is positively related to lung function and slower age-related decline in lung function (7). For vitamin C, β-carotene, and fiber, prospective and cross-sectional studies show that individuals with high intakes had better lung function than those with low intakes (8–13), whereas less consistent findings are observed for other antioxidants, including vitamin E (10, 11, 14, 15).

Growing evidence supports the hypothesis that dietary flavonoids may also have a beneficial effect on lung function. Experimental studies in animals have found specific flavonoids to attenuate airway hyperresponsiveness, inflammation, and remodeling (16–19). Cross-sectional findings from 2 population-based studies in adults also observed positive associations between dietary flavan-3-ol and flavonol intake with forced expiratory volume in 1 s (FEV_1_)^10^ and with forced vital capacity (FVC), respectively (20, 21). To our knowledge, no longitudinal studies to date have evaluated if specific flavonoid subclasses are associated with slower rates of age-related lung function decline.

In addition, no studies to our knowledge have examined the impact of dietary anthocyanin intake on lung function. Anthocyanins are a subclass of flavonoids derived from fruit, predominantly berries, which have been shown to be associated with lower risk of hypertension and myocardial infarction (22, 23) and with lower levels of inflammation (24). Findings from acute and short-term clinical trials also suggest beneficial effects of both purified anthocyanins and intake of berries on a range of inflammatory biomarkers (25–27). The ingestion of anthocyanin-rich strawberries and fruit juice has also been shown to improve antioxidant enzyme activities and plasma antioxidant capacity in experimental human studies, providing evidence of beneficial effects on oxidative stress, another key mechanism involved in lung function (28, 29).

Given the previous findings observed for flavonoid intake with lung function (20, 21), and for flavonoid intake with inflammation and oxidative stress biomarkers (24, 28, 29), we hypothesized that a higher habitual intake of anthocyanins, flavonols, flavan-3-ols, and their polymeric forms would be associated with beneficial changes in lung function. We investigated if dietary intake of the major flavonoid subclasses was associated with a slower age-related decline in lung function in a community-based cohort of elderly men living in the Boston area.

## METHODS

Study participants included in this longitudinal analysis were enrolled in the VA (Veterans Affairs) Normative Aging Study, an ongoing longitudinal study of aging established in 1963, details of which have been published previously (30). Briefly, the Normative Aging Study is a closed cohort of 2280 male volunteers from the Greater Boston area aged 21–80 y at entry, who enrolled after an initial health screening determined that they were free of known chronic medical conditions. Participants have undergone detailed examinations every 3–5 y, including routine physical examinations, laboratory tests, collection of medical history information, and completion of questionnaires on smoking history, food intake, and other factors that may influence health, which were confirmed by a trained interviewer. Physical examinations included measurement of height and lung function (FEV_1_ and FVC). Pulmonary disorders confirmed by a physician (asthma, chronic bronchitis, or emphysema) and smoking history were collected through the American Thoracic Society questionnaire (31). The present study was approved by the human research committees of the Harvard School of Public Health and the Veterans Affairs Boston Health care System, and written informed consent was obtained from subjects before participation.

For the present analysis, there were 1281 eligible participants with at least 1 visit between May 1992 and October 2008, when computations of dietary flavonoid intake were available for all self-administered semi-quantitative food-frequency questionnaires (FFQs). Of those eligible, there were 1048 participants with at least 1 visit with data available on spirometry, dietary flavonoid intake, and covariates of interest (**Supplemental Figure 1**). This analysis included 839 participants whose lung function was measured and who completed an FFQ in at least 2 visits (*n* = 2623 visits); there were 287 participants with 2 visits, 262 with 3 visits, 187 with 4 visits, and 103 with 5 visits. Visits with implausible values for total energy intake for men (<800 or >4200 kcal) were excluded from the analysis. For additional description of the study participation, please refer to the Methods section of the **Supplemental Material**.

### Outcome assessment

Pulmonary function tests were performed as previously reported (32), and acceptability of spirograms was judged according to American Thoracic Society standards. Briefly, a water-filled recording spirometer was used to obtain measures of FEV_1_ (mL) and FVC (mL), with values adjusted by body temperature and ambient pressure. These spirometric tests were performed in accordance with American Thoracic Society guidelines (33, 34). In this study, approximate normal distributions were observed for both FEV_1_ and FVC.

### Dietary assessment

Since May of 1992, the average daily dietary intakes of food and beverage items were assessed with a self-administered, validated, semi-quantitative FFQ adapted from the questionnaire used in the Nurses’ Health Study. Details on the reproducibility and validity of this FFQ for estimating daily nutrient intakes were published elsewhere (35, 36). A database for assessment of intake of the different flavonoid subclasses was constructed as previously described (22) and was based on the updated and expanded USDA flavonoid content of foods and the proanthocyanidin databases (37, 38) together with other sources. Intakes of individual compounds were calculated as the sum of the consumption frequency of each food multiplied by the content of the specific flavonoid for the specified portion size. We derived intakes (mg/d) of the subclasses commonly consumed in the US diet, specifically anthocyanins (cyanidin, delphinidin, malvidin, pelargonidin, petunidin, peonidin), flavanones (eriodictyol, hesperetin, naringenin), flavan-3-ols (catechins, gallocatechins, epicatechin, epigallocatechin, epicatechin-3-gallate, epigallocatechin-3-gallate), flavonols (quercetin, kaempferol, myricetin, isohamnetin), flavones (luteolin, apigenin), and polymers (including proanthocyanidins excluding monomers, theaflavins, and thearubigins). Refer to the Supplemental Material for additional details describing the FFQ and computation of flavonoid subclasses.

### Statistical analysis

All statistical analyses were carried out by using SAS version 9.3 (SAS Institute). Utilizing concurrent measures of lung function, flavonoid intake, and covariates, we used time-varying linear mixed-effects regression models with random participant-specific intercepts, which account for the correlation of repeated measures (39), to model continuous FEV_1_ and FVC as a function of major subclass of flavonoid intake. Each flavonoid subclass was characterized in quartiles (lowest quartile as reference) and was fit in a separate model and adjusted for age at first visit, height (cm), total energy intake (kcal/d, in quartiles), and time since first visit; we estimated the adjusted difference in annual change in lung function over time associated with flavonoid intake via an interaction between each flavonoid quartile (lowest quartile as reference) and time since first visit in the model.

All of the models were further adjusted for nondietary and dietary factors that were ascertained at each visit, including the following: race (black or white as reference), smoking status (current smoker, recent quitter, longtime quitter, or never smoker as reference), cumulative pack-years smoked, physician diagnosis of chronic bronchitis or asthma or emphysema (no as reference), use of medication for asthma (no as reference), use of statins (no as reference), years of education (<12, 12, 13–15, >15 y as reference), percentage of census tract below poverty level, total fruit intake (servings/d, in quartiles), total vegetable intake (servings/d, in quartiles), and physical activity (metabolic equivalent tasks/wk, in quartiles). We evaluated effect modification by smoking status (ever or never), physician-diagnosed obstructive lung disease (asthma, chronic bronchitis, or emphysema), and obesity [BMI (in kg/m^2^) ≥30 or <30]. Three-way interaction terms between flavonoid subclass intake, time since first visit, and the modifying variables were tested separately to evaluate whether the potential effect modifiers underlie susceptibility to the exposure response.

In addition, we adjusted for the intake of hot or cold cereal (servings/d), dark fish (servings/wk), processed meats (servings/d), caffeine (mg/d), vitamin C (without supplements, mg/d), omega-3 fatty acids (g/d), total dietary fiber intake (g/d), and for the presence of comorbid conditions including hypertension, coronary heart disease, and diabetes mellitus. We also conducted food-based analyses of the main sources of each flavonoid subclass for all FFQs collected between 1992 and 2004 (*n* = 3366). Because healthier men may be more likely to participate in a subsequent follow-up visits, we also applied stabilized inverse probability weights to correct for this potential survival bias (40) in all models. Refer to the Supplemental Material for additional description of the computation of stabilized inverse probability weights.

## RESULTS

Characteristics of the participants by yearly average anthocyanin intake measured at first visit are presented in **Table 1**. The median age of all of the participants at the first visit was 66.8 y, with an age range of 49–92 y. Participants with higher anthocyanin intakes smoked less, were more physically active, and consumed more fruit and vegetables. The mean follow-up time for all participants was 7.4 y, and the maximum follow-up time was 16 y. The distributions of FEV_1_ and FVC levels among all participants at each visit are summarized in **Supplemental Table 1**.

**TABLE 1:** Characteristics of participants at first visit by quartiles of total anthocyanin intake^1^

	Anthocyanin intake [median (IQR)]			
Characteristics	Quartile 1: 1.1 (0.5, 1.6) mg/d	Quartile 2: 3.6 (2.8, 4.4) mg/d	Quartile 3: 12.7 (8.0, 13.7) mg/d	Quartile 4: 21.1 (16.5, 27.4) mg/d
Participants, *n*	211	208	210	210
FEV_1_, mL	2747.4 ± 668.6^2^	2789.2 ± 630.4	2838.1 ± 628.1	2893.4 ± 634.6
FVC, mL	3681.8 ± 764.9	3760.3 ± 735.5	3787.5 ± 742.1	3850.8 ± 754.7
Age, y	65.8 ± 7.1	67.6 ± 7.0	67.1 ± 6.5	66.8 ± 6.7
Height, cm	1730.7 ± 68.9	1736.0 ± 71.9	1737.2 ± 64.3	1743.0 ± 65.6
Smoking status, *n* (%)				
Current smokers	20 (9.5)	14 (6.7)	7 (3.3)	6 (2.9)
Recent quitters (<10 y)	30 (14.2)	27 (13.0)	21 (10.0)	20 (9.5)
Longtime quitters (≥10 y)	107 (50.7)	104 (50.0)	122 (58.1)	117 (55.7)
Never smokers	54 (25.6)	63 (30.3)	60 (28.6)	67 (31.9)
Cumulative pack-years	27.0 ± 29.2	19.6 ± 22.4	20.9 ± 28.2	16.2 ± 22.1
Race, *n* (%)				
Black	1 (0.5)	6 (2.9)	5 (2.4)	1 (0.5)
White	210 (99.5)	202 (97.1)	205 (97.6)	209 (99.5)
Years of education	14.1 ± 2.9	14.5 ± 2.8	15.4 ± 2.8	15.5 ± 2.9
Percentage below poverty level in census tract	6.4 ± 5.5	6.8 (5.9)	5.8 (5.1)	5.7 (4.7)
Physician-diagnosed asthma, *n* (%)	14 (6.6)	9 (4.3)	12 (5.7)	6 (2.9)
Physician-diagnosed chronic bronchitis, *n* (%)	14 (6.6)	12 (5.8)	16 (7.6)	10 (4.8)
Physician-diagnosed emphysema, *n* (%)	8 (3.8)	11 (5.3)	14 (6.7)	8 (3.8)
Use of asthma medications, *n* (%)	6 (2.8)	13 (6.3)	7 (3.3)	5 (2.4)
Use of statins, *n* (%)	29 (13.7)	29 (13.9)	29 (13.8)	29 (13.8)
Metabolic equivalent tasks/wk	11.4 ± 13.4	18.5 ± 24.7	20.2 ± 25.4	22.2 ± 27.6
Total energy intake, kcal/d	1678.3 ± 568.7	2005.7 ± 592.0	2060.3 ± 567.9	2249.1 ± 651.4
Total fruit intake, servings/d	1.7 ± 1.2	2.6 ± 1.5	2.7 ± 1.5	3.6 ± 2.1
Total vegetable intake, servings/d	2.4 ± 1.6	3.2 ± 1.7	3.5 ± 1.9	4.3 ± 2.3
Total dietary fiber intake,^3^ mg/d	15.5 ± 6.2	20.6 ± 6.5	22.4 ± 8.7	25.4 ± 9.0
Hot and cold cereal intake, servings/d	0.4 ± 0.4	0.5 ± 0.4	0.5 ± 0.4	0.6 ± 0.6
Vitamin C intake (without supplements), mg/d	121.6 ± 70.0	169.1 ± 88.6	169.4 ± 70.8	209.9 ± 115.1
Caffeine intake, mg/d	249.3 ± 191.2	250.7 ± 194.5	231.8 ± 186.5	207.1 ± 178.6
n–3 Intake,^4^ g/d	0.3 ± 0.3	0.3 ± 0.3	0.3 ± 0.2	0.4 ± 0.4
Dark fish intake, servings/d	0.0 ± 0.1	0.1 ± 0.1	0.1 ± 0.1	0.1 ± 0.1
Other fish intake, servings/d	0.1 ± 0.1	0.1 ± 0.2	0.1 ± 0.1	0.1 ± 0.1
Cold cuts, servings/d	0.2 ± 0.4	0.2 ± 0.2	0.2 ± 0.4	0.1 ± 0.2
Flavanones, mg/d	44.8 ± 45.0	59.3 ± 46.3	56.8 ± 39.9	65.2 ± 43.9
Flavan-3-ols, mg/d	45.2 ± 59.1	52.7 ± 60.1	51.7 ± 58.5	60.3 ± 61.7
Flavonols, mg/d	14.8 ± 10.0	18.6 ± 10.9	19.4 ± 10.9	24.7 ± 12.3
Flavones, mg/d	1.8 ± 1.5	2.5 ± 1.5	2.6 ± 1.3	3.4 ± 1.8
Polymers, mg/d	154.3 ± 193.9	207.0 ± 204.8	213.5 ± 207.0	267.0 ± 207.3
Hypertension, *n* (%)	125 (59.2)	125 (60.1)	120 (57.1)	117 (55.7)
Coronary heart disease, *n* (%)	41 (19.4)	39 (18.8)	37 (17.6)	39 (18.6)
Diabetes mellitus, *n* (%)	34 (16.1)	30 (14.4)	26 (12.4)	23 (11.0)
Obesity (BMI ≥30 kg/m^2^), *n* (%)	59 (28.0)	55 (26.4)	49 (23.3)	49 (23.3)

1 *n* = 839. Quartile distribution of anthocyanin intake was derived from intake estimated at first visit; the median (IQR) for year of first visit was 1994 (1993, 1996). FEV_1_, forced expiratory volume in 1 s; FVC, forced vital capacity.

2 Unadjusted mean ± SD (all such values).

3 Excluding 160 participants with a first visit in 1992 when estimation of total fiber intake was not available; numbers of participants in quartiles 1, 2, 3, and 4 are 173, 164, 167, and 175, respectively.

4 Includes EPA and DHA, no α-linolenic acid.

**FIGURE 1:**
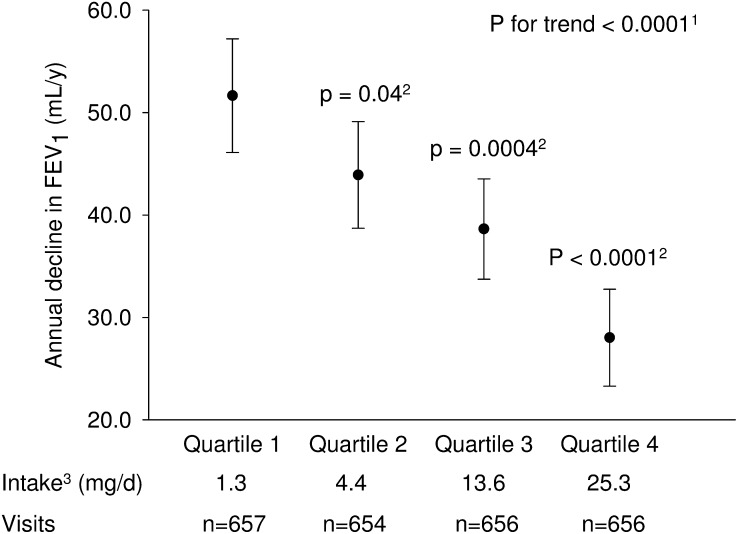
Adjusted mean (95% CI) annual decline in FEV_1_ by quartile of total anthocyanin intake. The annual FEV_1_ decline for each quartile of anthocyanin intake was estimated in a linear mixed regression model of FEV_1_ with a random intercept for study participant and adjusting for the following covariates: time since first visit, age at first visit, height, race, presence of obstructive lung diseases, asthma medication use, statin medication use, education, percentage of census tract below poverty level, smoking status, pack-years of smoking, physical activity, total energy intake, total fruit intake, total vegetable intake, and 2-way interaction terms between time since first visit and each quartile of anthocyanin intake; the variable coefficient for time since first visit reflects the adjusted mean annual FEV_1_ decline for quartile of anthocyanin intake that is assigned the reference category. ^1^The *P* value for test of linear trend across quartile categories of anthocyanin intake was based on a linear mixed regression model in which the value of median intake was assigned to each category of anthocyanin intake; this quartile median variable was used as a continuous measure in the regression model. ^2^*P* values for quartiles 2, 3, and 4 are for the *F* test comparison with quartile 1. ^3^Median values are presented for each quartile category of anthocyanin intake. FEV_1_, forced expiratory volume in 1 s.

**FIGURE 2:**
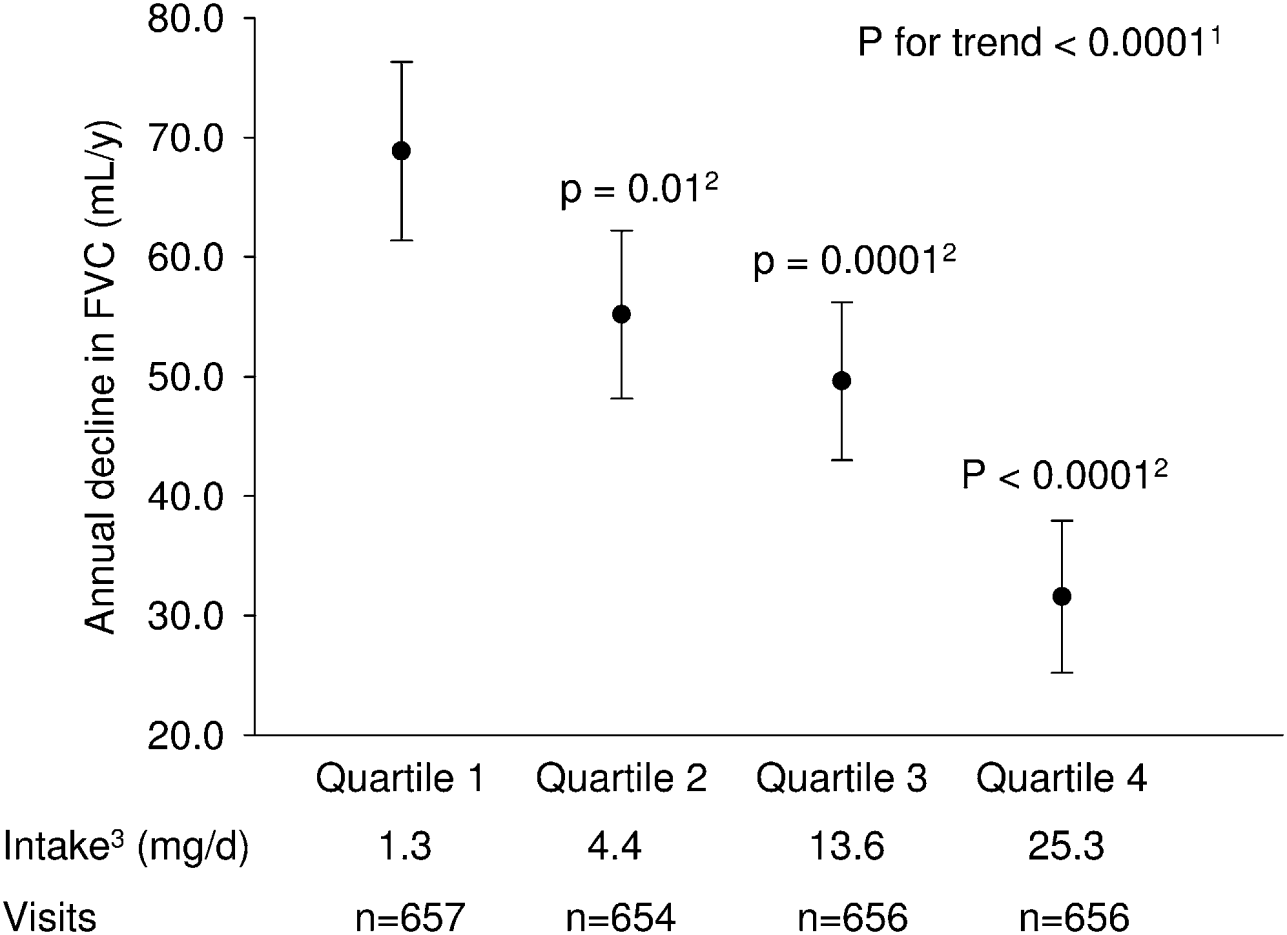
Adjusted mean (95% CI) annual decline in FVC by quartile of total anthocyanin intake. The annual FVC decline for each quartile of anthocyanin intake was estimated in a linear mixed regression model of FVC with a random intercept for study participant and adjusting for the following covariates: time since first visit, age at first visit, height, race, presence of obstructive lung diseases, asthma medication use, statin medication use, education, percentage of census tract below poverty level, smoking status, pack-years of smoking, physical activity, total energy intake, total fruit intake, total vegetable intake, and 2-way interaction terms between time since first visit and each quartile of anthocyanin intake; the variable coefficient for time since first visit reflects the adjusted mean annual FVC decline for quartile of anthocyanin intake that is assigned the reference category. ^1^The *P* value for test of linear trend across quartile categories of anthocyanin intake was based on a linear mixed regression model in which the value of median intake was assigned to each category of anthocyanin intake; this quartile median variable was used as a continuous measure in the regression model. ^2^*P* values for quartiles 2, 3, and 4 are for the *F* test comparison with quartile 1. ^3^Median values are presented for each quartile category of anthocyanin intake. FVC, forced vital capacity.

After multivariate adjustment for a range of lifestyle and dietary factors, higher total anthocyanin intake significantly (*P* < 0.05) attenuated the rates of age-related annual decline in FEV_1_ and FVC (**Figures 1** and **2**, respectively). Compared with participants in the first quartile, participants in the fourth quartile of anthocyanin intake had a slower rate of FEV_1_ decline by 23.6 (95% CI, 16.6, 30.7) mL/y (Figure 1, **Table 2**). The linear trend for adjusted difference in annual change in FEV_1_ across quartiles of increasing anthocyanin intake was also significant (*P*-trend < 0.0001). A similar pattern of association was also observed for FVC as shown in Figure 2. Compared with participants in the first quartile, participants in the fourth quartile of anthocyanin intake had a slower rate of decline in FVC by 37.3 (95% CI: 27.8, 46.8) mL/y (*P*-trend < 0.0001) (Figure 2, **Table 3**). There was little difference in the observed associations for anthocyanin intake between the minimal and fully adjusted models (Tables 2 and 3, respectively). Furthermore, the observed associations between anthocyanin intake and annual change in FEV_1_ and FVC were robust after the application of stabilized inverse probability weights.

**TABLE 2:** Adjusted differences in annual changes in FEV_1_ (mL/y) for yearly average anthocyanin, flavan-3-ol, and polymer subclass intakes^1^

	Adjusted mean annual change in FEV_1_	Adjusted difference in mean annual change in FEV_1_ relative to quartile 1			
	Quartile 1	Quartile 2	Quartile 3	Quartile 4	*P*-trend^2^
Anthocyanins					
Median intake, mg/d	1.3	4.4	13.6	25.3	—
Person-visits, *n*	657	654	656	656	—
Minimally adjusted model^3^	−51.4 ± 2.7^4^	7.6 (0.2, 15.2)^5^	12.3 (5.2, 19.5)	22.8 (15.8, 29.78)	<0.0001
Fully adjusted model^6^	−51.7 ± 2.8	7.8 (0.3, 15.2)	13.0 (5.8, 20.2)	23.6 (16.6, 30.7)	<0.0001
IPW model^7^	−52.0 ± 2.8	6.8 (−0.6, 13.7)	13.7 (6.6, 20.9)	22.9 (15.9, 29.9)	<0.0001
Flavan-3-ols					
Median intake, mg/d	9.7	21.4	45.7	99.3	—
Person-visits,* n*	656	655	656	656	—
Minimally adjusted model^3^	−41.1 ± 2.5	2.1 (−4.9, 9.0)	2.0 (−4.9, 9.0)	6.2 (−0.5, 12.9)	0.07
Fully adjusted model^6^	−41.2 ± 2.6	1.9 (−5.1, 8.9)	2.7 (−4.3, 9.6)	6.6 (−0.1, 13.3)	0.05
IPW model^7^	−42.2 ± 2.6	0.9 (−6.2, 7.9)	4.7 (−2.2, 11.6)	7.1 (0.4, 13.9)	0.02
Polymers					
Median intake, mg/d	46.6	109.3	201.6	440.6	—
Person-visits, *n*	656	655	656	656	—
Minimally adjusted model^3^	−41.2 ± 2.6	0.3 (−6.8, 7.4)	4.0 (−3.1, 11.1)	5.8 (−1.1, 12.6)	0.08
Fully adjusted model^6^	−41.3 ± 2.7	0.6 (−6.6, 7.7)	4.4 (−2.8, 11.5)	6.1 (−0.8, 13.0)	0.07
IPW model^7^	−42.3 ± 2.7	0.8 (−6.4, 8.0)	5.4 (−1.7, 12.5)	6.7 (−0.2, 13.6)	0.05

1 FEV_1_, forced expiratory volume in 1 s; IPW, inverse probability weight.

2 *P* values for the test of linear trend across quartile categories of flavonoid subclass intake were based on a linear mixed regression model with the value of median intake assigned to each quartile category of flavonoid subclass intake; this quartile median variable was used as a continuous measure in the regression model.

3 As estimated in linear mixed-effects regression model of FEV_1_ with a random intercept for study participant and adjusting for the following covariates: flavonoid intake (mg/d, in quartiles), time since first visit (y), age at first visit (y), height (cm), and total energy intake (kcal/d, in quartiles); β is the variable coefficient for the interaction between time since first visit and category of flavonoid subclass intake where quartile 1 is the reference category.

4 Mean ± SEM (all such values).

5 β 95% CI in parentheses (all such values).

6 The fully adjusted model includes additional adjustment for race (black or white as reference), smoking status (current smoker, recent quitter, longtime quitter, or never smoker as reference), cumulative pack-years smoked, physician diagnosis of chronic bronchitis or asthma or emphysema (no as reference), asthma medication use (no as reference), statin medication use (no as reference), years of education (<12, 12, 13–15, >15 y as reference), percentage of census tract below poverty level, total fruit intake (servings/d, in quartiles), total vegetable intake (servings/d, in quartiles), and physical activity (metabolic equivalent tasks/wk, in quartiles).

7 The IPW model is the fully adjusted model after application of stabilized inverse weights for censoring by loss to follow-up.

**TABLE 3:** Adjusted differences in annual change in FVC (mL/y) yearly average anthocyanin, flavan-3-ol, and polymer subclass intakes^1^

		Adjusted difference in mean annual change in FVC relative to quartile 1			
	Adjusted mean annual change in FVC: quartile 1	Quartile 2	Quartile 3	Quartile 4	*P*-trend^2^
Anthocyanins					
Median intake, mg/d	1.3	4.4	13.6	25.3	
Person-visits, *n*	657	654	656	656	
Minimally adjusted model^3^	−69.5 ± 3.7^4^	13.7 (3.7, 23.8)^5^	18.7 (9.0, 28.4)	36.5 (27.0, 45.9)	<0.0001
Fully adjusted model^6^	−68.9 ± 3.8	13.7 (3.6, 23.7)	19.2 (9.5, 28.9)	37.3 (27.8, 46.8)	<0.0001
IPW model^7^	−68.4 ± 3.7	11.2 (1.2, 21.2)	19.2 (9.6, 28.8)	35.6 (26.2, 45.0)	<0.0001
Flavan-3-ols					
Median intake, mg/d	9.7	21.4	45.7	99.3	
Person-visits, *n*	656	655	656	656	
Minimally adjusted model^3^	−51.3 ± 3.3	3.2 (−6.3, 12.7)	−2.4 (−11.8, 7.1)	8.4 (−0.8, 17.5)	0.09
Fully adjusted model^6^	−50.3 ± 3.5	2.4 (−7.0, 11.9)	−2.0 (−11.4, 7.4)	8.6 (−0.5, 17.8)	0.06
IPW model^7^	−51.4 ± 3.5	0.6 (−8.9, 10.0)	1.2 (−8.2, 10.5)	9.5 (0.4, 18.5)	0.02
Polymers					
Median intake, mg/d	46.6	109.3	201.6	440.6	
Person-visits, *n*	656	655	656	656	
Minimally adjusted model^3^	−52.4 ± 3.5	0.7 (−8.9, 10.4)	3.8 (−5.8, 13.5)	8.5 (−0.8, 17.8)	0.05
Fully adjusted model^6^	−51.3 ± 3.7	0.5 (−9.2, 10.1)	3.9 (−5.7, 13.6)	8.4 (−0.9, 17.7)	0.05
IPW model^7^	−52.8 ± 3.7	0.7 (−9.1, 10.4)	6.3 (−3.4, 15.9)	9.5 (0.2, 18.8)	0.03

1 FVC, forced vital capacity; IPW, inverse probability weight.

2 *P* values for the test of linear trend across quartile categories of flavonoid subclass intake were based on a linear mixed regression model with the value of median intake assigned to each quartile category flavonoid subclass intake; this quartile median variable was used as a continuous measure in the regression model.

3 As estimated in linear mixed-effects regression model of FVC with a random intercept for study participant and adjusting for the following covariates: flavonoid intake (mg/d, in quartiles), time since first visit (y), age at first visit (y), height (cm), and total energy intake (kcal/d, in quartiles); β is the variable coefficient for the interaction between time since first visit and category of flavonoid subclass intake where quartile 1 is the reference category.

4 Mean ± SEM (all such values).

5 β 95% CI in parentheses (all such values).

6 The fully adjusted model includes additional adjustment for race (black, white as reference), smoking status (current smoker, recent quitter, longtime quitter, or never smoker as reference), cumulative pack-years smoked, physician diagnosis of chronic bronchitis or asthma or emphysema (no as reference), asthma medication use (no as reference), statin medication use (no as reference), years of education (<12, 12, 13–15, >15 y as reference), percentage of census tract below poverty level, total fruit intake (servings/d, in quartiles), total vegetable intake (servings/d, in quartiles), and physical activity (metabolic equivalent tasks/wk, in quartiles).

7 The IPW model is the fully adjusted model after application of stabilized inverse weights for censoring by loss to follow-up.

To a lesser extent, an attenuation of lung function decline was also observed for intake of flavan-3-ols and polymers in the highest quartile. After application of stabilized inverse probability weights, participants in the fourth quartile of flavan-3-ol intake had a significantly slower rate of decline in FEV_1_ and FVC by 7.1 (95% CI: 0.4, 13.9) and 9.5 (95% CI: 0.4, 18.5) mL/y, respectively (*P*-trend = 0.02) compared with participants in the lowest quartile (Tables 2 and 3, respectively). A positive association of similar magnitude was observed for polymer intake in the fourth quartile and annual change in FVC (*P*-trend = 0.03) (Table 3).

The results for the other flavonoid subclasses are presented in the **Supplemental Tables 2 **and** 3**. No significant associations were observed for flavonol and flavone subclasses. Significant negative associations were observed between the second and fourth quartiles of flavonone intake and annual change in FVC, indicating faster decline in FVC in association with flavonone intake (Supplemental Table 3); in contrast, a positive association of similar magnitude was observed for flavonone intake in the second quartile and a linear trend was not observed. However, the observed changes in FVC for the second and fourth quartiles of flavonone intake were reduced moderately after the application of stabilized inverse probability weights and were nonsignificant.

After stratification by smoking status, slower rates of annual decline in FEV_1_ and FVC in association with total anthocyanin intake were observed in both current/former smokers and in never smokers (**Supplemental Table 4**). Although the slowest rates of decline in association with anthocyanin intake were found in never smokers, a 3-way interaction between smoking status, anthocyanin intake, and time since first visit on lung function was not significant. Similarly, no interactions were observed between anthocyanin intake and the presence of obstructive lung diseases and obesity (data not shown). The adjusted differences in annual lung function decline for anthocyanin intake remained robust after additional individual adjustment for intakes of total dietary fiber, hot or cold breakfast cereal, vitamin C, caffeine, n–3 fatty acids, fish, and processed meats and for the presence of comorbid conditions including hypertension, coronary heart disease, and diabetes mellitus (**Supplemental Tables 5 **and** 6**).

The results of food-based analyses utilizing all FFQs collected between 1992 and 2004 indicate that intakes of blueberries (49.9%), red wine (17.6%), and strawberries (15.5%) contributed most to overall anthocyanin intake. To confirm our findings for total anthocyanin intake and to relate the effects on lung function decline to public health and dietary guidelines, we examined associations between weekly intakes of blueberries, red wine, and strawberries and annual change in lung function. Blueberry intake was associated with the slowest rate of annual decline in lung function; compared with no or very low intake, consuming ≥2 servings of blueberries/wk was associated with a slower rate of decline in FEV_1_ and FVC by 22.5 (95% CI: 10.5, 34.2) and 37.6 (95% CI: 21.8, 53.4) mL/y, respectively (*P*-trend < 0.0001) (**Table 4**). To a lesser extent, similar findings were observed for strawberry intake. No significant associations were observed for red wine intake (data not shown).

**TABLE 4:** Adjusted differences in mean annual change in lung function (mL/y) for blueberry and strawberry intakes^1^

	Mean annual change	Adjusted difference in mean annual change			
	No intake or <1 serving/mo	<1 serving/wk	1 serving/wk	≥2 servings/wk	*P*-trend^2^
Blueberries					
Person-visits, *n*	1596	723	196	108	
FEV_1_	−41.5 ± 1.7^3^	2.9 (−2.7, 8.4)^4^	17.0 (7.7, 26.3)	22.5 (10.8, 34.2)	<0.0001
FVC	−53.1 ± 2.3	7.0 (−0.5, 14.5)	18.5 (6.0, 31.1)	37.9 (22.1, 53.7)	<0.0001
Strawberries					
Person-visits, *n*	1188	963	304	168	
FEV_1_	−42.8 ± 2.0	4.8 (−0.6, 10.3)	14.5 (6.6, 22.5)	13.1 (3.8, 22.4)	<0.0001
FVC	−51.4 ± 2.7	7.7 (0.3, 15.1)	16.1 (5.3, 26.1)	19.7 (7.0, 32.3)	<0.0001

1 As estimated in linear mixed regression models of FEV_1_ (and FVC) with a random intercept for study participant and adjusting for the following covariates: time since first visit, age at first visit, height, race, smoking status, cumulative pack-years smoked, physician diagnosis of chronic bronchitis or asthma or emphysema, asthma medication use, statin use, years of education, percentage of census tract below poverty level, total energy intake, total fruit intake, total vegetable intake, and physical activity. β is the variable coefficient for the interaction between time since first visit and category of blueberry and strawberry intake where no intake or <1 serving/mo is the reference category. FEV_1_, forced expiratory volume in 1 s; FVC, forced vital capacity.

2 *P* values for the test of linear trend across categories of blueberry (and strawberry) intake were based on a linear mixed regression model where the ordinal variable for blueberry (and strawberry) intake was used as a continuous measure.

3 Mean ± SEM (all such values).

4 β 95% CI in parentheses (all such values).

## DISCUSSION

Our findings suggest that habitual anthocyanin intake was associated with slower age-related decline in FEV_1_ and FVC, independent of established dietary and nondietary risk factors. In addition, an attenuation of lung function decline associated with anthocyanin intake was stronger in never smokers than in ever smokers. To a lesser extent, an attenuation of lung function decline was also observed for intakes of flavan-3-ols and polymers in the highest quartile. The beneficial changes in lung function over time associated with anthocyanin intake complement findings from recent studies that suggest protective effects of these compounds on a number of chronic diseases including cardiovascular disease and associated risk factors (22–24, 41).

Anthocyanins are present in red/blue fruits and may, in part, explain the previously reported population-based evidence that showed that fruit consumption was positively related to lung function and protective against the incidence of chronic nonspecific lung diseases (7, 42, 43). In food-based analyses of the main anthocyanin sources, an attenuation of lung function decline was also observed for frequent intakes of blueberries and strawberries. The beneficial changes in lung function associated with frequent consumption of berries may possibly exceed the decline in lung function attributable to smoking, assuming that moderate- to-heavy smoking men have, on average, a 15-mL/y larger decline in FEV_1_ than do nonsmokers (44). The beneficial change in FEV_1_ associated with the highest quartile of anthocyanin intake is within the range of a previously reported association between vitamin C intake and reduction in decline in FEV_1_ (50.8 mL/y; 95% CI: 3.8, 97.9 mL/y) from a general population–based study in the United Kingdom (8).

The present study provides novel evidence that suggests that berries may contain specific constituents that may attenuate lung function decline. The addition of total dietary fiber, vitamin C, and other dietary constituents, including total fruit and total vegetable intake, to our multivariate model did not substantially influence the observed associations between anthocyanin intake and longitudinal change in lung function. These findings suggest that the benefits of anthocyanins are specific and not simply reflective of those who eat a high-plant-based diet.

A growing body of evidence from experimental studies supports a role for anthocyanins and their degradation products/microbial metabolites on mechanisms involved in lung function. The microbiome is likely to play a key role in the bioactivity of anthocyanins because degradation is swift after intake and the microbially derived metabolites are present in the circulation longer and at higher concentrations than the parent anthocyanins (45–48). Recent findings from in vitro studies suggest that nutritionally relevant amounts of these downstream metabolites exert a greater anti-inflammatory effect than the parent molecules (49, 50), although the impact on airway inflammation is unknown. Previous studies also showed that anthocyanins, including anthocyanin-rich bilberry extract, can inhibit the activation of nuclear transcription factor κB (51–53) and attenuate oxidative stress through activations of the Nuclear factor, erythroid 2-like 2 (Nrf2) signaling pathway (54). In a recent cross-sectional analysis in 2375 Framingham Heart Study Offspring Cohort participants, habitual intakes of both total anthocyanins and strawberries were inversely associated with a combined inflammatory score, which integrated a range of inflammatory and oxidative stress biomarkers (24). In the limited experimental human studies conducted to date, berry intake was also associated with improved markers of oxidative stress including cell antioxidant defense against DNA damage (28, 29, 55, 56).

Only 2 previous observational studies (20, 21) examined associations between flavonoid intake and lung function. These studies used earlier flavonoid databases that covered only a few subclasses and did not include anthocyanins or flavanones. When combining the intakes of flavan-3-ol, flavonol, and flavone subclasses, a 44-mL higher difference in FEV_1_ level was observed when comparing extreme intake quintiles (117 compared with 15 mg/d) in a large cross-sectional analysis in 13,651 adults in the Netherlands (20). Similarly, investigators in Chile observed 100- and 70-mL differences in FVC level when comparing highest to lowest quintiles of flavonol and flavan-3-ol intakes, respectively, in a cross-sectional analysis in 1232 young adults (21). Although we hypothesized that higher intakes of flavones, flavonols, flavan-3-ols, and their polymeric forms would also attenuate lung function decline over time, no associations were observed for flavone and flavonol intake in the present study. However, a slower decline in FEV_1_ and FVC over time was also observed with a high intake of flavan-3-ols. A similar finding was also observed for a high intake of polymers and FVC decline.

The strengths of this study include the longitudinal design with multiple repeat measures of dietary flavonoid intake and lung function to evaluate longitudinal change, a large sample size with long-term follow-up, adjustment for multiple confounders, and methods to address selection bias. Our databases also allowed us to comprehensively assess the range of flavonoids present in the habitual diet. However, there are a number of limitations to consider. Our findings relate to predominantly elderly white men; thus, the generalizability to women and younger and other racial-ethnic population groups requires further study. Although we adjusted for possible confounders that are known to be associated with lung function, there remains the possibility of residual confounding with respect to these factors or unmeasured confounding from additional unmeasured factors. However, given our detailed and updated adjustment for potential confounders, it is unlikely that these would account fully for the associations we observed.

Average daily flavonoid subclass intakes were calculated from a database developed by using recent USDA databases with additional input from European Union sources (EuroFIR eBASIS; http://www.eurofir.org), allowing us to robustly assess intakes using the best available databases at the time. Flavonoid content also varies depending on a number of factors, including growing conditions, processing, and storage; however, despite these variations, these data allowed us to rank participants and compare low and high intakes in large population groups. Although our FFQ has not been specifically validated for intakes of flavonoid subclasses, correlations between the main dietary sources of flavonoids (fruit, tea, vegetables, and wine) have been determined for our FFQ (57, 58) and flavonoid biomarkers are correlated with intakes of fruit and vegetables (59). Current research is moving closer to identifying biomarkers of anthocyanin intake (46), but it is possible that our findings for anthocyanins might be due to other constituents found in the foods that contribute most to this subclass. However, even additional adjustment of other potentially beneficial constituents of fruit, including vitamin C and fiber, did not substantially attenuate the relation between anthocyanins and lung function, suggesting that anthocyanins may be another important dietary constituent. In an observational cohort study such as the VA Normative Aging Study, it is impossible to disentangle the relative influence of all of the constituents of plant-based foods.

In summary, we observed that a higher dietary intake of anthocyanins was associated with an attenuation of lung function decline in this longitudinal sample of predominantly elderly white men. The present findings suggest a potential role for anthocyanins in berry fruit in slowing age-related decline in lung function. Further prospective studies are needed to confirm these novel associations.

## Footnotes

10 Abbreviations used: FEV_1_, forced expiratory volume in 1 s; FFQ, food-frequency questionnaire; FVC, forced vital capacity; Nrf2, Nuclear factor, erythroid 2-like 2.

## References

[1] Fletcher C, Peto R, Tinker CM, Speizer FE The natural history of chronic bronchitis and emphysema. Oxford (United Kingdom): Oxford University Press; 1976.

[2] Rijcken B, Weiss ST Longitudinal analyses of air-way responsiveness and pulmonary function decline. Am J Respir Crit Care Med 1996;154:S246–9. 8970396 10.1164/ajrccm/154.6_Pt_2.S246

[3] Neas LM, Schwartz J Pulmonary function levels as predictors of mortality in a national sample of US adults. Am J Epidemiol 1998;147:1011–8. 9620044 10.1093/oxfordjournals.aje.a009394

[4] Sin DD, Wu L, Man SF The relationship between reduced lung function and cardiovascular mortality: a population-based study and a systematic review of the literature. Chest 2005;127:1952–9. 15947307 10.1378/chest.127.6.1952

[5] Mannino DM, Davis KJ Lung function decline and outcomes in an elderly population. Thorax 2006;61:472–7. 16517577 10.1136/thx.2005.052449 PMC2111202

[6] Mannino DM, Buist AS Global burden of COPD: risk factors, prevalence and future trends. Lancet 2007;370:765–73. 17765526 10.1016/S0140-6736(07)61380-4

[7] Eisner MD, Anthonisen N, Coultas D, Kuenzli N, Perez-Padilla R, Postma D, Romieu I, Silverman EK, Balmes JR; Committee on Nonsmoking COPD, Environmental and Occupational Health Assembly. An official American Thoracic Society public policy statement: novel risk factors and the global burden of chronic obstructive pulmonary disease. Am J Respir Crit Care Med 2010;182:693–718. 20802169 10.1164/rccm.200811-1757ST

[8] McKeever TM, Scrivener S, Broadfield E, Jones Z, Britton J, Lewis SA Prospective study of diet and decline in lung function in a general population. Am J Respir Crit Care Med 2002;165:1299–303. 11991883 10.1164/rccm.2109030

[9] Schwartz J, Weiss ST Relationship between dietary vitamin C intake and pulmonary function in the First National Health and Nutrition Examination Survey (NHANES I). Am J Clin Nutr 1994;59:110–4. 8279390 10.1093/ajcn/59.1.110

[10] Hu G, Cassano PA Antioxidant nutrients and pulmonary function: the Third National Health and Nutrition Examination Survey (NHANES III). Am J Epidemiol 2000;151:975–81. 10853636 10.1093/oxfordjournals.aje.a010141

[11] Guénégou A, Leynaert B, Pin I, Le Moël G, Zureik M, Neukirch F Serum carotenoids, vitamins A and E, and 8 year lung function decline in a general population. Thorax 2006;61:320–6. 16565267 10.1136/thx.2005.047373 PMC2104600

[12] Bentley AR, Kritchevsky SB, Harris TB, Holvoet P, Jensen RL, Newman AB, Lee JS, Yende S, Bauer D, Cassano PA Dietary antioxidants and forced expiratory volume in 1 s decline: the Health, Aging and Body Composition Study. Eur Respir J 2012;39:979–84. 22005919 10.1183/09031936.00190010 PMC3390780

[13] Kan H, Stevens J, Heiss G, Rose KM, London SJ Dietary fiber, lung function, and chronic obstructive pulmonary disease in the atherosclerosis risk in communities study. Am J Epidemiol 2008;167:570–8. 18063592 10.1093/aje/kwm343 PMC2377022

[14] Cassano PA, Guertin KA, Kristal AR, Ritchie KE, Bertoia ML, Arnold KB, Crowley JJ, Hartline J, Goodman PJ, Tangen CM, A randomized controlled trial of vitamin E and selenium on rate of decline in lung function. Respir Res 2015;16:35. 25889509 10.1186/s12931-015-0195-5 PMC4404242

[15] Hanson C, Lyden E, Furtado J, Campos H, Sparrow D, Vokonas P, Litonjua AA Serum tocopherol levels and vitamin E intake are associated with lung function in the Normative Aging Study. Clin Nutr. In press. 10.1016/j.clnu.2015.01.020 PMC4529394 25715694

[16] Rogerio AP, Dora CL, Andrade EL, Chaves JS, Silva LF, Lemos-Senna E, Calixto JB Anti-inflammatory effect of quercetin-loaded microemulsion in the airways allergic inflammatory model in mice. Pharmacol Res 2010;61:288–97. 19892018 10.1016/j.phrs.2009.10.005

[17] Yang T, Luo F, Shen Y, An J, Li X, Liu X, Ying B, Liao Z, Dong J, Guo L, Quercetin attenuates airway inflammation and mucus production induced by cigarette smoke in rats. Int Immunopharmacol 2012;13:73–81. 22465384 10.1016/j.intimp.2012.03.006

[18] Taguchi L, Pinheiro NM, Olivo CR, Choqueta-Toledo A, Grecco SS, Lopes FD, Caperuto LC, Martins MA, Tiberio IF, Câmara NO, A flavanone from Baccharis retusa (Asteraceae) prevents elastase-induced emphysema in mice by regulating NF-κB, oxidative stress and metalloproteinases. Respir Res 2015;16:79. 26122092 10.1186/s12931-015-0233-3 PMC4489216

[19] Toledo AC, Sakoda CP, Perini A, Pinheiro NM, Magalhães RM, Grecco S, Tibério IF, Câmara NO, Martins MA, Lago JH, Flavonone treatment reverses airway inflammation and remodelling in an asthma murine model. Br J Pharmacol 2013;168:1736–49. 23170811 10.1111/bph.12062 PMC3605879

[20] Tabak C, Smit HA, Heederik D, Ocke MC, Kromhout D Diet and chronic obstructive pulmonary disease: independent beneficial effects of fruits, whole grains, and alcohol (the MORGEN Study). Clin Exp Allergy 2001;31:747–55. 11422134 10.1046/j.1365-2222.2001.01064.x

[21] Garcia-Larsen V, Amigo H, Bustos P, Bakolis I, Rona RJ Ventilatory function in young adults and dietary antioxidant intake. Nutrients 2015;7:2879–96. 25884660 10.3390/nu7042879 PMC4425179

[22] Cassidy A, O’Reilly EJ, Kay C, Sampson L, Franz M, Forman J, Curhan G, Rimm EB Habitual intake of flavonoid subclasses and incident hypertension in adults. Am J Clin Nutr 2011;93:338–47. 21106916 10.3945/ajcn.110.006783 PMC3021426

[23] Cassidy A, Mukamal KJ, Liu L, Franz M, Eliassen AH, Rimm EB High anthocyanin intake is associated with a reduced risk of myocardial infarction in young and middle-aged women. Circulation 2013;127:188–96. 23319811 10.1161/CIRCULATIONAHA.112.122408 PMC3762447

[24] Cassidy A, Rogers G, Peterson JJ, Dwyer JT, Lin H, Jacques PF Higher dietary anthocyanin and flavonol intakes are associated with anti-inflammatory effects in a population of US adults. Am J Clin Nutr 2015;102:172–81. 26016863 10.3945/ajcn.115.108555 PMC4480670

[25] Zhu Y, Ling W, Guo H, Song F, Ye Q, Zou T, Li D, Zhang Y, Li G, Xiao Y, Anti-inflammatory effect of purified dietary anthocyanin in adults with hypercholesterolemia: a randomized controlled trial. Nutr Metab Cardiovasc Dis 2013;23:843–9. 22906565 10.1016/j.numecd.2012.06.005

[26] Edirisinghe I, Banaszewski K, Cappozzo J, Sandhya K, Ellis CL, Tadapaneni R, Kappagoda CT, Burton-Freeman BM Strawberry anthocyanin and its association with postprandial inflammation and insulin. Br J Nutr 2011;106:913–22. 21736853 10.1017/S0007114511001176

[27] Moazen S, Amani R, Homayouni Rad A, Shahbazian H, Ahmadi K, Taha Jalali M. Effects of freeze-dried strawberry supplementation on metabolic biomarkers of atherosclerosis in subjects with type 2 diabetes: a randomized double-blind controlled trial. Ann Nutr Metab 2013;63:256–64. 24334868 10.1159/000356053

[28] Alvarez-Suarez JM, Giampieri F, Tulipani S, Casoli T, Di Stefano G, González-Paramás AM, Santos-Buelga C, Busco F, Quiles JL, Cordero MD, One-month strawberry-rich anthocyanin supplementation ameliorates cardiovascular risk, oxidative stress markers and platelet activation in humans. J Nutr Biochem 2014;25:289–94. 24406274 10.1016/j.jnutbio.2013.11.002

[29] Kuntz S, Kunz C, Herrmann J, Borsch CH, Abel G, Fröhling B, Dietrich H, Rudloff S Anthocyanins from fruit juices improve the antioxidant status of healthy young female volunteers without affecting anti-inflammatory parameters: results from the randomised, double-blind, placebo-controlled, cross-over ANTHONIA (ANTHOcyanins in Nutrition Investigation Alliance) study. Br J Nutr 2014;112:925–36. 25089359 10.1017/S0007114514001482

[30] Bell B, Rose C, Damon A The Normative Aging Study: an interdisciplinary and longitudinal study of health and aging. Aging Hum Dev 1972;3:5–17.

[31] Ferris BG Epidemiology Standardization Project (American Thoracic Society). Am Rev Respir Dis 1978;118:1–120. 742764

[32] Sparrow D, O’Connor G, Colton T, Barry CL, Weiss ST The relationship of nonspecific bronchial responsiveness to the occurrence of respiratory symptoms and decreased levels of pulmonary function. The Normative Aging Study. Am Rev Respir Dis 1987;135:1255–60. 3296893 10.1164/arrd.1987.135.6.1255

[33] American Thoracic Society. Standardization of spirometry—1987 update. Statement of the American Thoracic Society. Am Rev Respir Dis 1987;136:1285–98. 3674589 10.1164/ajrccm/136.5.1285

[34] American Thoracic Society. Standardization of spirometry, 1994 update. Am J Respir Crit Care Med 1995;152:1107–36. 7663792 10.1164/ajrccm.152.3.7663792

[35] Willett WC, Sampson L, Stampfer MJ, Rosner B, Bain C, Witschi J, Hennekens CH, Speizer FE Reproducibility and validity of a semiquantitative food frequency questionnaire. Am J Epidemiol 1985;122:51–65. 4014201 10.1093/oxfordjournals.aje.a114086

[36] Rimm EB, Giovannucci EL, Stampfer MJ, Colditz GA, Litin LB, Willett WC Reproducibility and validity of an expanded self-administered semiquantitative food frequency questionnaire among male health professionals. Am J Epidemiol 1992;135:1114–26. 1632423 10.1093/oxfordjournals.aje.a116211

[37] USDA. USDA Database for the Flavonoid Content of Selected Foods. Release 2.1. Washington (DC): USDA; 2007.

[38] USDA. USDA Database for the Proanthocyanidin Content of Selected Foods. Washington (DC): USDA; 2004.

[39] Fitzmaurice GM, Laird NM, Ware JH Applied longitudinal analysis. Hoboken (NJ): Wiley-Interscience; 2004.

[40] Cole SR, Hernán MA Constructing inverse probability weights for marginal structural models. Am J Epidemiol 2008;168:656–64. 18682488 10.1093/aje/kwn164 PMC2732954

[41] Wedick NM, Pan A, Cassidy A, Rimm EB, Sampson L, Rosner B, Willett W, Hu FB, Sun Q, van Dam RM Dietary flavonoid intakes and risk of type 2 diabetes in US men and women. Am J Clin Nutr 2012;95:925–33. 22357723 10.3945/ajcn.111.028894 PMC3302366

[42] Carey IM, Strachan DP, Cook DG Effects of changes in fresh fruit consumption on ventilatory function in healthy British adults. Am J Respir Crit Care Med 1998;158:728–33. 9730997 10.1164/ajrccm.158.3.9712065

[43] Miedema I, Feskens EJ, Heederik D, Kromhout D Dietary determinants of long-term incidence of chronic nonspecific lung diseases. The Zutphen Study. Am J Epidemiol 1993;138:37–45. 8333425 10.1093/oxfordjournals.aje.a116775

[44] Kerstjens HA, Rijcken B, Schouten JP, Postma DS Decline of FEV1 by age and smoking status: facts, figures, and fallacies. Thorax 1997;52:820–7. 9371217 10.1136/thx.52.9.820 PMC1758654

[45] Williamson G, Clifford MN Colonic metabolites of berry polyphenols: the missing link to biological activity? Br J Nutr 2010;104(Suppl 3):S48–66. 20955650 10.1017/S0007114510003946

[46] Czank C, Cassidy A, Zhang Q, Morrison DJ, Preston T, Kroon PA, Botting NP, , Kay CD Human metabolism and elimination of the anthocyanin, cyanidin-3-glucoside: a (13)C-tracer study. Am J Clin Nutr 2013;97:995–1003. 23604435 10.3945/ajcn.112.049247

[47] de Ferrars RM, Czank C, Zhang Q, Botting NP, Kroon PA, Cassidy A, Kay CD The pharmacokinetics of anthocyanins and their metabolites in humans. Br J Pharmacol 2014;171:3268–82. 24602005 10.1111/bph.12676 PMC4080980

[48] Faria A, Fernandes I, Norberto S, Mateus N, Calhau C. Interplay between anthocyanins and gut microbiota. J Agric Food Chem 2014;62:6898–902. 24915058 10.1021/jf501808a

[49] Amin HP, Czank C, Raheem S, Zhang Q, Botting NP, Cassidy A, Kay CD Anthocyanins and their physiologically relevant metabolites alter the expression of IL-6 and VCAM-1 in CD40L and oxidized LDL challenged vascular endothelial cells. Mol Nutr Food Res 2015;59:1095–106. 25787755 10.1002/mnfr.201400803 PMC4950056

[50] di Gesso JL, Kerr JS, Zhang Q, Raheem S, Yalamanchili SK, O'Hagan D, Kay CD, O'Connell MA Flavonoid metabolites reduce tumor necrosis factor-α secretion to a greater extent than their precursor compounds in human THP-1 monocytes. Mol Nutr Food Res 2015;59:1143–54. 25801720 10.1002/mnfr.201400799 PMC4973837

[51] Min SW, Ryu SN, Kim DH Anti-inflammatory effects of black rice, cyanidin-3-O-beta-D-glycoside, and its metabolites, cyanidin and protocatechuic acid. Int Immunopharmacol 2010;10:959–66. 20669401 10.1016/j.intimp.2010.05.009

[52] Hamalainen M, Nieminen R, Vuorela P, Heinonen M, Moilanen E Anti-inflammatory effects of flavonoids: genistein, kaempferol, quercetin, and daidzein inhibit STAT-1 and NF-kappaB activations, whereas flavone, isorhamnetin, naringenin, and pelargonidin inhibit only NFkappaB activation along with their inhibitory effect on iNOS expression and NO production in activated macrophages. Mediators Inflamm 2007;2007:45673. 10.1155/2007/45673 PMC2220047 18274639

[53] Luo H, Lv XD, Wang GE, Li YF, Kurihara H, He RR Anti-inflammatory effects of anthocyanins-rich extract from bilberry (Vaccinium myrtillus L.) on croton oil-induced ear edema and Propionibacterium acnes plus LPS-induced liver damage in mice. Int J Food Sci Nutr 2014;65:594–601. 24548119 10.3109/09637486.2014.886184

[54] Thoppil RJ, Bhatia D, Barnes KF, Haznagy-Radnai E, Hohmann J, Darvesh AS, Bishayee A Black currant anthocyanins abrogate oxidative stress through Nrf2- mediated antioxidant mechanisms in a rat model of hepatocellular carcinoma. Curr Cancer Drug Targets 2012;12:1244–57. 22873220 10.2174/156800912803987968

[55] Riso P, Klimis-Zacas D, Del Bo’ C, Martini D, Campolo J, Vendrame S, Møller P, Loft S, De Maria R, Porrini M Effect of a wild blueberry (Vaccinium angustifolium) drink intervention on markers of oxidative stress, inflammation and endothelial function in humans with cardiovascular risk factors. Eur J Nutr 2013;52:949–61. 22733001 10.1007/s00394-012-0402-9

[56] Del B C, Riso P, Campolo J, Møller P, Loft S, Klimis-Zacas D, Brambilla A, Rizzolo A, Porrini M A single portion of blueberry (Vaccinium corymbosum L) improves protection against DNA damage but not vascular function in healthy male volunteers. Nutr Res 2013;33:220–7. 23507228 10.1016/j.nutres.2012.12.009

[57] Salvini S, Hunter DJ, Sampson L, Stampfer MJ, Colditz GA, Rosner B, Willett WC Food-based validation of a dietary questionnaire: the effects of week-to-week variation in food consumption. Int J Epidemiol 1989;18:858–67. 2621022 10.1093/ije/18.4.858

[58] Feskanich D, Rimm EB, Giovannucci EL, Colditz GA, Stampfer MJ, Litin LB, Willett WC Reproducibility and validity of food intake measurements from a semiquantitative food frequency questionnaire. J Am Diet Assoc 1993;93:790–6. 8320406 10.1016/0002-8223(93)91754-e

[59] Krogholm KS, Bysted A, Brantsaeter AL, Jakobsen J, Rasmussen SE, Kristoffersen L, Toft U Evaluation of flavonoids and enterolactone in overnight urine as intake biomarkers of fruits, vegetables and beverages in the Inter99 cohort study using the method of triads. Br J Nutr 2012;108:1904–12. 22453033 10.1017/S0007114512000104

